# The Nitrite of Amyl in Epilepsy

**Published:** 1874-02

**Authors:** 


### The Nitrite of Amyl in Epilepsy.

Dr. Crichton Browne has contributed to the last volume of "West Riding Lunatic Asylum Medical Reports" a paper in which he describes his experience in the use of nitrite of amyl in epilepsy. The results at which he has arrived are mentioned in the *Medical Press and Circular:*—

Being engaged in tracing out the areas of blushing as induced by nitrite of amyl in different individuals and under different circumstances, with a view to elucidate the laws regulating the diffusion of that form of emotional expression; Dr. Crichton Browne was struck by the fact that the degree and extent to which the blushing caused by nitrite of amyl is manifested are influenced by certain pathological states. He found that general paralytic patients may inhale a considerable amount without displaying any marked flushing, even of the face, and that epileptics cannot breathe the smallest quantity without exhibiting extreme cutaneous hyperæmia over the face, neck, and chest. Guided by these observations and by an ingenious argument founded upon them, he was led to conclude that if the nitrite of amyl could be given immediately before an epileptic fit, the spasm of vessels might be prevented, and so the whole sequence of morbid events averted. "And," as he forcibly remarks, "a fit averted in epilepsy is no slight gain; it is, in fact, a step made towards recovery, and a postponement of those degenerative consequences which are, as a rule, developed in proportion to the frequency and severity of the fits. To interrupt a pathological habit is to give a chance of recovery; to control the fits is to limit the destructiveness of epilepsy." In several cases in which the nitrite of amyl was administered immediately after an aura the usual fit did not supervene, and in one case in which it was administered regularly three times a day, a series of fits from which the patient was suffering was abruptly interrupted. In rabbits, too, rendered artificially epileptic by Professor Ferrier, it was noted that the fit which invariably followed on electrical irritation applied to the exposed brain when no interference took place, was arrested by the inhalation of nitrite of amyl. "The result of all my experiments is to convince me," says Dr. Crichton Browne, "that it will be found invaluable in many cases in not only postponing but in altogether preventing epileptic seizures. The utility of an agent possessing this power can scarcely be exaggerated. It will, I believe, supersede other methods of attempting to avert the fit by acting upon indications afforded by the aura. Pressure upon, or ligature of a limb, section of a nerve trunk, or cauterization of the surface from which an aura originates have done good service in certain cases, in hindering the accession of seizures, but the nitrite of amyl appears to be a more ready and certain means for compassing the same end. A vinaigrette small stoppered bottle containing a sponge soaked in nitrite of amyl and carried in the pocket, so as to be at hand on the occurrence of an aura, will, I think, be found a safeguard to many sufferers from epilepsy. Whenever there is time after the initiation of the aura, and before the development of the proper phenomena of the fit, to breathe the nitrite of amyl freely, the fit, with its terrible accompaniments and disastrous sequelæ, may, in many instances, be not merely postponed but abolished."

But there is another epoch in epilepsy besides the pause between the aura and the fit, when according to Dr. Crichton Browne's experience, the nitrite of amyl may prove beneficial, that is, at an advanced stage, when the alarming condition, the *status epilepticus*, is developed. In ten cases of the *status epilepticus* the nitrite of amyl has been used, and eight of these have terminated in recovery. Under its influence several patients have rallied from what was apparently a hopeless condition. Whenever it is inhaled the breathing becomes freer, the circulation is relieved, and the seizures are diminished in frequency and severity. It appears to act with a directness and certainty that cannot be ascribed to any other remedy hitherto employed in the *status epilecticus.—Medical and Surgical Reporter*.

